# Assessing Attitudes Towards Medicinal Cannabis and Cannabinoid Use Among Dementia Patients and Their Caregivers

**DOI:** 10.1002/alz70858_100668

**Published:** 2025-12-25

**Authors:** Alana Vanessa Kerr, Jacqueline Dossetor, Jody Huynh, Yun Tae Hwang

**Affiliations:** ^1^ Brain and Mind Centre, University of Sydney, Sydney, NSW, Australia

## Abstract

**Background:**

Dementia is a broad term that covers multiple conditions which affect the brain, such as Alzheimer's disease and Frontotemporal dementia. The collection of symptoms caused by these disorders vary and can include disrupted mood, apathy, agitation, aggression, disinhibition, and/or sleep. There has been increasing interest in exploring potential benefits of cannabis‐derived medicine in alleviating neuropsychiatric symptoms of dementia. We examined the attitudes towards the use of cannabinoids for symptomatic treatment of dementia among Australian adults diagnosed with different dementia types, their caregivers, and healthy controls.

**Method:**

Patients with a diagnosis of dementia, their caregivers, and healthy controls from the FRONTIER research database, at the University of Sydney, completed an online questionnaire assessing attitudes toward medicinal cannabis and cannabinoid use. The questionnaire inquired about the perceived benefits and risks of cannabis use; experience with cannabis use; and interest in participating in a hypothetical clinical trial using medicinal cannabis for the symptomatic treatment of dementia. There were 77 respondents to the questionnaire: 22 dementia patients, 30 study partners, and 25 healthy controls.

**Result:**

Of respondents with a diagnosis of dementia, 73% said they would consider participating in a clinical trial exploring the potential therapeutic effects of medicinal cannabis. Out of all participants who completed the survey, 80% said they would consider participating in such a trial. While only 24% of the participants reported previous recreational or medicinal cannabis use, there remains a strong general interest in exploring the potential benefits of cannabinoids through clinical trials.

**Conclusion:**

The results from this questionnaire show that there is a strong interest from Australian dementia patients and their caregivers to participate in future clinical trials that would assess the effectiveness of medicinal cannabis in alleviating dementia symptoms. As evidenced by this and other studies, there is growing support for the consideration of cannabis‐derived products as medical treatments. We propose that robustly designed clinical trials with clearly defined outcome measures would be of interest and have the potential to expand symptomatic treatment options to those living with dementia.

